# Physiologically based pharmacokinetic-pharmacodynamic evaluation of meropenem in CKD and hemodialysis individuals

**DOI:** 10.3389/fphar.2023.1126714

**Published:** 2023-03-07

**Authors:** Guoliang Deng, Fan Yang, Ning Sun, Danhong Liang, Anfen Cen, Chen Zhang, Suiqin Ni

**Affiliations:** ^1^ School of Biology and Biological Engineering, South China University of Technology, Guangzhou, Guangdong, China; ^2^ Department of Pharmacy, The Second Affiliated Hospital, School of Medicine, South China University of Technology, Guangzhou, Guangdong, China; ^3^ Department of Hepatobiliary Surgery, Guangzhou Eighth People’s Hospital, Guangzhou, Guangdong, China; ^4^ Guangzhou First People’s Hospital, School of Medicine, South China University of Technology, Guangzhou, Guangdong, China

**Keywords:** meropenem, physiologically based pharmacokinetic model, probability, CKD, hemodialysis, pharmacodynamics

## Abstract

**Objective:** Chronic kidney disease (CKD) has significant effects on renal clearance of drugs. The application of antibiotics in CKD patients to achieve the desired therapeutic effect is challenging. This study aims to determine meropenem plasma exposure in the CKD population and further investigate optimal dosing regimens.

**Methods:** A healthy adult PBPK model was established using the meropenem’s physicochemical parameters, pharmacokinetic parameters, and available clinical data, and it was scaled to the populations with CKD and dialysis. The differences between the predicted concentration, C_max_, and AUC_last_ predicted and observed model values were assessed by mean relative deviations (MRD) and geometric mean fold errors (GMFE) values and plotting the goodness of fit plot to evaluate the model’s performance. Finally, dose recommendations for CKD and hemodialysis populations were performed by Monte Carlo simulations.

**Results:** The PBPK models of meropenem in healthy, CKD, and hemodialysis populations were successfully established. The MRD values of the predicted concentration and the GMFE values of C_max_ and AUC_last_ were within 0.5–2.0-fold of the observed data. The simulation results of the PBPK model showed the increase in meropenem exposure with declining kidney function in CKD populations. The dosing regimen of meropenem needs to be further adjusted according to the renal function of CKD patients. In patients receiving hemodialysis, since meropenem declined more rapidly during the on-dialysis session than the off-dialysis session, pharmacodynamic evaluations were performed for two periods separately, and respective optimal dosing regimens were determined.

**Conclusion:** The established PBPK model successfully predicted meropenem pharmacokinetics in patients with CKD and hemodialysis and could further be used to optimize dosing recommendations, providing a reference for personalized clinical medication.

## 1 Introduction

In recent years, the incidence of chronic kidney disease (CKD) has increased rapidly. CKD has become a critical problem affecting global public health because of its high incidence, high mortality, high medical expenses, and low awareness rate (Global Burden of Disease Study, 2015; Ene-Iordache et al., 2016). With impaired renal function, GFR decreased accordingly. When the GFR falls below 15 mL/min, patients enter stage 5 or develop end-stage renal disease (ESRD). At this time, the remaining renal function is insufficient to sustain life, and renal replacement therapy (RRT, including hemodialysis, peritoneal dialysis, and kidney transplantation) is required to maintain the body’s regular needs (Huang et al., 2022). Of note, due to the impaired quality of their humoral and cellular immune responses, CKD patients, including those receiving dialysis, are particularly vulnerable to infection (Aloy et al., 2020). Bloodstream infections have become the leading cause of morbidity and mortality in these patients (Fisher et al., 2020).

Understanding the pharmacokinetic changes of anti-infective agents is crucial in CKD and dialysis patients. Infections in patients with CKD and dialysis are usually nosocomial, and the common pathogens are *Pseudomonas aeruginosa* and *Escherichia coli*, most of which are drug-resistant bacteria. In treating drug-resistant bacteria, carbapenems such as meropenem and high-grade antibiotics are used clinically (Alshaer et al., 2022). Meropenem is a carbapenem antibiotic widely used to treat infections caused by multidrug-resistant Gram-negative bacilli. Meropenem is also recommended for the treatment of peritoneal dialysis-associated peritonitis (PDAP) (Li et al., 2022). Regarding the elimination of meropenem, it is primarily cleared renally by both glomerular filtration and renal tubular secretion. About 70% of drugs are excreted in the urine as prototypes (Harrison et al., 1993). Most of the remaining drug is metabolized to inactive metabolites by renal dehydrogenase 1 (DHP1 or DPEP1) on proximal tubular epithelial cells (Shibayama et al., 2007). Early and cumulative target attainment significantly impacts the treatment outcome for nosocomial infections. Carbapenem exposure should be optimized as early as possible and maintained throughout treatment. As a drug excreted through the kidney, the change in renal function in patients may affect the blood concentration of meropenem and the therapeutic effect and even lead to adverse drug reactions. In addition, during hemodialysis, the pharmacokinetic parameters of meropenem can change significantly, and the use of hemodialysis has additional profound effects on drug clearance (Rubino et al., 2018; Saito et al., 2020). There is currently inadequate information regarding the use of meropenem in patients on dialysis (Pfizer Laboratories Div Pfizer Inc, 2019).

Recent FDA guidance on renal impairment advocates physiology-based pharmacokinetic (PBPK) model to early characterize the effect of renal impairment on drug pharmacokinetics (US Food Drug Admin, 2020). Therefore, the PBPK model can be a tool for an in-depth understanding of the pharmacokinetic changes of meropenem in CKD and HD patients. In recent years, the pharmacokinetic/pharmacodynamic (PK/PD) model combined with Monte Carlo simulations (MCS) has been developed to comprehensively consider the minimum inhibitory concentration (MIC) distribution of specific bacteria and pharmacokinetic variation of patients without clinical outcome research. It can provide an essential reference for evaluating, comparing, and optimizing drug administration regimens (Roberts et al., 2011).

This study aims to investigate the pharmacokinetics of meropenem in CKD and ESRD patients undergoing hemodialysis and design safe therapeutic dose regimens for this patient population by using PBPK model combined with pharmacodynamic evaluation.

## 2 Materials and methods

### 2.1 Software

The meropenem PBPK model was developed using PK-Sim^®^ and MoBi^®^ (Open Systems Pharmacology Suite 11.0, www.open-systems-pharmacology.org). Model parameter optimization using the Monte Carlo algorithm and sensitivity analysis were performed in PK-Sim^®^. WebPlotDigitizer (Version 4.5) was used to digitize published clinical research data. Non-compartmental PK analysis of the plasma concentration-time data was performed using the PKSolver program (Zhang et al., 2010). Dosing regimens were simulated by Oracle Crystal Ball (Ver 11.1.2.4) combined with PK parameters.

### 2.2 Clinical data

Plasma concentration-time profiles of meropenem were gathered and digitized from published clinical studies in healthy individuals, CKD and dialysis populations. Detailed information is provided in Supplementary Tables S1, S2 in the Electronic Supplementary Material (ESM). Clinical studies of healthy volunteers were used for early meropenem PBPK model construction, divided into a training dataset for model construction and a test dataset for model evaluation. Concentration–time profiles for CKD and the hemodialysis PBPK model test datasets were selected from studies that (1) included data from different periods of CKD and (2) provided different dialysis parameter settings.

### 2.3 Development of PBPK models for meropenem in healthy individuals

The development of meropenem PBPK model in healthy individuals is stepwise. We collected all available information on meropenem ADME properties for the initial model construction. The physiological database built into PK-Sim^®^ provides various anatomical and physiological information (ICRP, 2002; Kuepfer et al., 2016). Some of these scalable parameters, such as organ volumes, the hematocrit value, and blood flows, will be adjusted based on the input demographics, while others will use default settings. In the development of the initial model, we mainly considered the renal excretion of meropenem. The excretion of meropenem involves glomerular filtration and renal tubular secretion. In general, renal tubular secretion involves two steps. It has been pointed out that OAT1 and OAT3 in the basolateral membrane of renal tubular cells distributed in the proximal kidney (Shibayama et al., 2007) and NPT1 in the apical membrane (Uchino et al., 2000) mainly mediate the renal tubular secretion of meropenem. The former extracts drugs (substrates) from the blood into epithelial cells, while the latter discharges intracellular drugs into the lumen for excretion (Zou et al., 2021). Considering the limited OAT1-mediated uptake (Shibayama et al., 2007) and the non-unique identification of the model, we hypothesized that the absorption of the basolateral membrane of renal proximal tubule cells is simply mediated by OAT3, while NPT1 will be at the apical side of renal tubules to explain the tubule secretion.

After building the initial model, we carried out multi-parameter synchronous optimization through parameter identification in PK-Sim^®^ to identify uncertain parameters. Monte Carlo algorithm was used to find the optimal solution of the parameters in a specific range to minimize the residuals between the simulation output and the actual observed values. In order to reduce the uncertainty, we collected the fractions excreted to urine of meropenem in clinical studies. In addition, to better distinguish the respective contribution of transporters in the basal lateral membrane and apical membrane of renal proximal tubular cells to renal tubular secretion, the concentration-time curve of meropenem after the addition of probenecid was simulated, compared with the control arm. As the potent clinical organic anion transporter (OAT) inhibitor, probenecid could inhibit OAT3 activity, while NPT1 is not affected. The probenecid model applied was developed by Britz et al. (2020). After the PBPK models were developed, we validated the developed PBPK model by comparing the simulated plasma concentration-time curves with the corresponding clinical studies.

### 2.4 CKD model development

For CKD modeling, we applied CKD pathophysiological changes to the healthy individual model to make it suitable for CKD patients, as described by Malik et al. (2020). We randomly generated 1,000 virtual populations in CKD-stage 3 to 5 for CKD population simulation, and the eGFR was set as 31–60 (ml/min/1.73 m^2^) in stage 3, 16–30 (ml/min/1.73 m^2^) in stage 4 and 1–15 (ml/min/1.73 m^2^) in stage 5. Based on the “intact nephron hypothesis (INH)", we hypothesized that renal secretion is reduced proportionally to the impaired GFR, as the ratio of eGFR_CKD_ to eGFR_nomal_ (Hanke et al., 2020). Thus, the adjustment method for the transporter concentrations in CKD virtual population was calculated according to Equation 1:

$$Scaling\;factor\;\left(transporter\;concentration\right)=\frac{{eGFR}_{CKD}}{{eGFR}_{nomal}}$$ (1)

where eGFR_nomal_ was set at 106.78 mL/min/1.73 m^2^. This value was obtained by creating a virtual adult male with average age (30 years), height (176 cm) and weight (73 kg) in PK-Sim^®^.

The non-renal clearance (CLNR) of meropenem is mainly mediated by DPEP1. We implemented the changes described by Sayama et al. (2014) in the CKD population, where DPEP1 concentration levels decreased to 69% in stage 3 CKD and to 64% in stage 4 and 5 CKD.

### 2.5 Development of hemodialysis model

For hemodialysis model construction, we imported the established CKD model into MOBI^®^ for dialyzer compartment expansion and connected it to arterial and venous blood compartments (Fuhr et al., 2020). The clearance rate of the hemodialysis model was determined by Eq. 2 (Michaels, 1966):

$$CLHD=BFR\left[\frac{e^{\left[\frac{KoA}{BFR}\left(1-\frac{BFR}{DFR}\right)\right]}-1}{e^{\left[\frac{KoA}{BFR}\left(1-\frac{BFR}{DFR}\right)\right]}-\frac{BFR}{DFR}}\right]$$ (2)

CLHD is the clearance rate of dialysis, BFR is the blood flow rate, DFR is the dialysate flow rate, and KoA is the product of the mass transfer coefficient and membrane surface area of Dialyzers used in clinical studies. The KoA was estimated to be 188 mL/min (Lee et al., 2022). BFR, DFR, and the duration of dialysis were adjusted according to the parameter settings of the clinical study.

### 2.6 PBPK model evaluation

Various methods were used to evaluate the performance of the meropenem PBPK model. First, the predicted concentration-time curves were compared with the data observed in their respective clinical studies. Additionally, a goodness-of-fit (GOF) plot was drawn to evaluate the agreement between the predicted values and their corresponding observed values, including plasma concentration C_max_ and area under the curve from the time of drug administration to the time of the last concentration measured concentration (AUC_last_) (Kuepfer et al., 2016). Further, overall performance was evaluated using commonly used metrics, Mean relative deviations (MRD) (Eq. 3) and Geometric mean fold errors (GMFE) (Eq. 4). Among them, MRD was used for quantitative evaluation of all plasma concentration predictions, and GMFE was used for all predicted AUC_last_ and C_max_ values. MRD and GMFE values ≤2 were interpreted as signs of adequate model performance (Jones et al., 2015).

$$MRD=10^{x};x=\sqrt{\frac{\sum_{i=1}^{m}{\left(\log_{10}c_{predicted,i}-\log_{10}c_{observed,i}\right)}^{2}}{m}}$$ (3)

where c_predicted, i_ = predicted plasma concentration, c_observed, i_ = observed plasma concentration, m = number of observed values.

$$GMFE=10^{x};x=\frac{\sum_{i=1}^{n}\left|\log_{10}\left.\left(\frac{predicted\;PK\;{parameter}_{i}}{observed\;PK\;{parameter}_{i}}\right)\right|\right.}{n}$$ (4)

where predicted PK parameter_i_ = predicted AUC_last_ or C_max_ value, observed PK parameter_i_ = observed AUC_last_ or C_max_value, n = number of studies.

To further ensure model reliability and to evaluate model performance, sensitivity analyses were performed. We calculated the sensitivity of meropenem predicted AUC_last_ to model parameters of a 500 mg dose of meropenem intravenous infusion over 30 min in healthy individuals, CKD and IHD PBPK models. Virtual adult males with mean demographic characteristics were created in PK-Sim, and eGFR was set to the median of the range of eGFR at different stages.

### 2.7 The dose regimen and PD evaluation of CKD and hemodialysis individuals

Oracle Crystal Ball (Ver 11.1.2.4) software was used to conduct a Monte Carlo simulation of meropenem administration plan of patients in different CKD stages and dialysis patients, with a sample size of 20,000. PK parameters for Monte Carlo simulation were calculated according to the simulation results of CKD and IHD population constructed above. Table 1 displays simulated dosing regimens ranging from a conventional dosing infusion (30 min) to a prolonged infusion (3 h) in different CKD stages. These dosing regimens were developed by Win et al. (2022) as well as our considerations. The simulated hemodialysis dosing regimen was further adjusted according to the results of the CKD simulation. The efficacy of meropenem is related to the time percentage of free drug concentration higher than MIC (*f* %T > MIC). When 40% *f* T > MIC, Meropenem demonstrated effective bactericidal activity (Nicolau, 2008). Recently it was shown that meropenem achieved better exposure at 100% *f* T > MIC (Veiga and Paiva, 2018). Therefore, 100% *f* T > MIC was used in this study as the pharmacodynamic target of CKD population simulation. Due to the rapid clearance of meropenem by hemodialysis, we chose 40% *f* T > MIC was evaluated as its pharmacodynamic target for IHD population simulation. The MIC sensitivity breakpoints in the simulations were set according to EUCAST breakpoint criteria (The European Committee on Antimicrobial Susceptibility Testing, 2022). The probability of target attainment (PTA) was calculated for various dosing regimens with different MICs. The optimal dosing regimen was considered when the PTA of a dosing regimen was greater than or equal to 90%. For the same PTA, the most convenient or lowest dose regimen was selected.

**TABLE 1:** Simulated dosing regimens in different CKD stages.

CKD - stage [eGFR (mL/min/1.73 m^2^)]	Total daily dose (g)	Dosing regimens	Infusion time
CKD - stage3 [31–60 (ml/min/1.73 m^2^)]	4	1000 mg q6h	30 min
			3 h
	3	1000 mg q8h	30 min
			3 h
	1.5	500 mg q8h	30 min
			3 h
CKD - stage4 [16–30 (ml/min/1.73 m^2^)]	3	1000 mg q8h	30 min
			3 h
	1.5	500 mg q8h	30 min
			3 h
CKD - stage5 [1–15 (ml/min/1.73 m^2^)]	3	1000 mg q8h	30 min
			3 h
	1.5	500 mg q8h	30 min
			3 h
	1	500 mg q12h	30min
			3 h
	1	1000 mg q24h	30 min
			3 h

## 3 Results

### 3.1 Development and validation of meropenem PBPK model in healthy individuals

**FIGURE 1:**
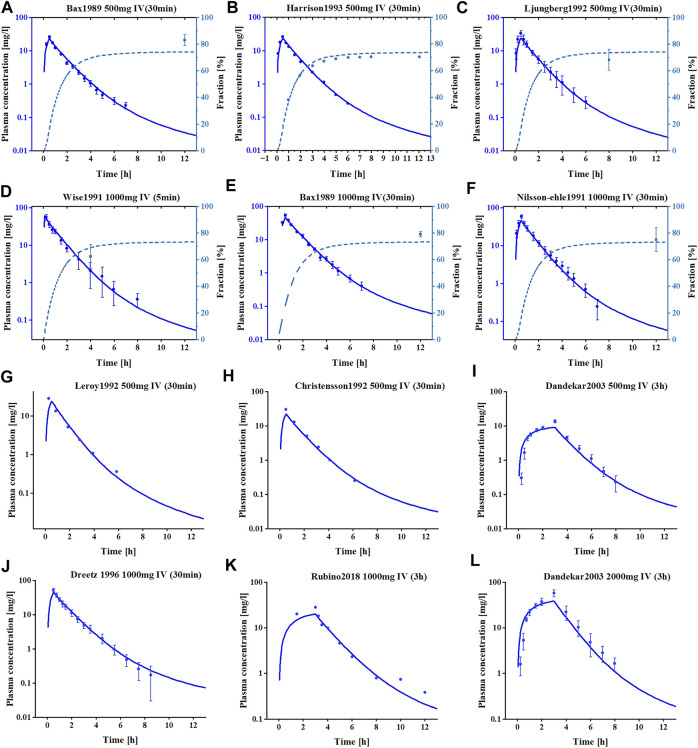
The simulated and observed plasma concentration-time profiles of meropenem PBPK model in healthy adults. **(A–F)** Are the plasma concentration-time profiles for the training datasets. **(G–L)** are the plasma concentration-time profiles for the test datasets. Clinical observed data are shown as mean values (circles), if available ±standard deviation (SD). Solid dark blue lines illustrate the predicted plasma concentrations, dashed light blue lines illustrate the predicted fractions excreted to urine.

The meropenem healthy individual model was built using a variety of dosages and infusion times and the final meropenem concentration-time profiles are shown in Figure 1 and Supplementary Figure S1. As shown, the established model is capable of accurately describing the distribution of meropenem plasma concentrations under different dosing regimens. Information on all clinical studies involved is provided in Supplementary Table S1 of the ESM. The input parameters for the final model are listed in Table 2 and the sensitivity analysis results of the parameters are provided in Supplementary Figure S2.

**TABLE 2:** Physiologically-based pharmacokinetic input parameters of meropenem.

Parameter	Values	Source
Molecular weight (g/mol)	383.46	Drugbank
pKa acid	3.47	Zhou et al. (2016)
pKa base	9.39	Chemaxon
Lipophilicity (logP)	1.25	Optimized
Water Solubility (mg/mL)	5.63	ALOGPS
Protein binding partner	Albumin	Drugbank
*f*_u_	98%	Drugbank
DPEP1 K_m_	3.56 mM	Park et al. (2002)
DPEP1 V_max_	79.34 μmol/l/min	Optimized
OAT3 K_m_	847 μM	Park et al. (2002); Shibayama et al. (2007)
OAT3 V_max_	18,156.72 μmol/l/min	Optimized
NPT1 K_m_	755.89 μM	Optimized
NPT1 V_max_	57.64 μmol/l/min	Optimized
GFR Fraction	1.00	Bax et al. (1989)

Abbreviations: *f*_u_, fraction unbound; DPEP1, dehydropeptidase 1 or renal dipeptidase 1; K_m_, Michaelis–Menten constant; V_max_, maximum rate of reaction; OAT3, organic anion transporter 3; NPT1, sodium-dependent phosphate transport protein 1.

**FIGURE 2:**
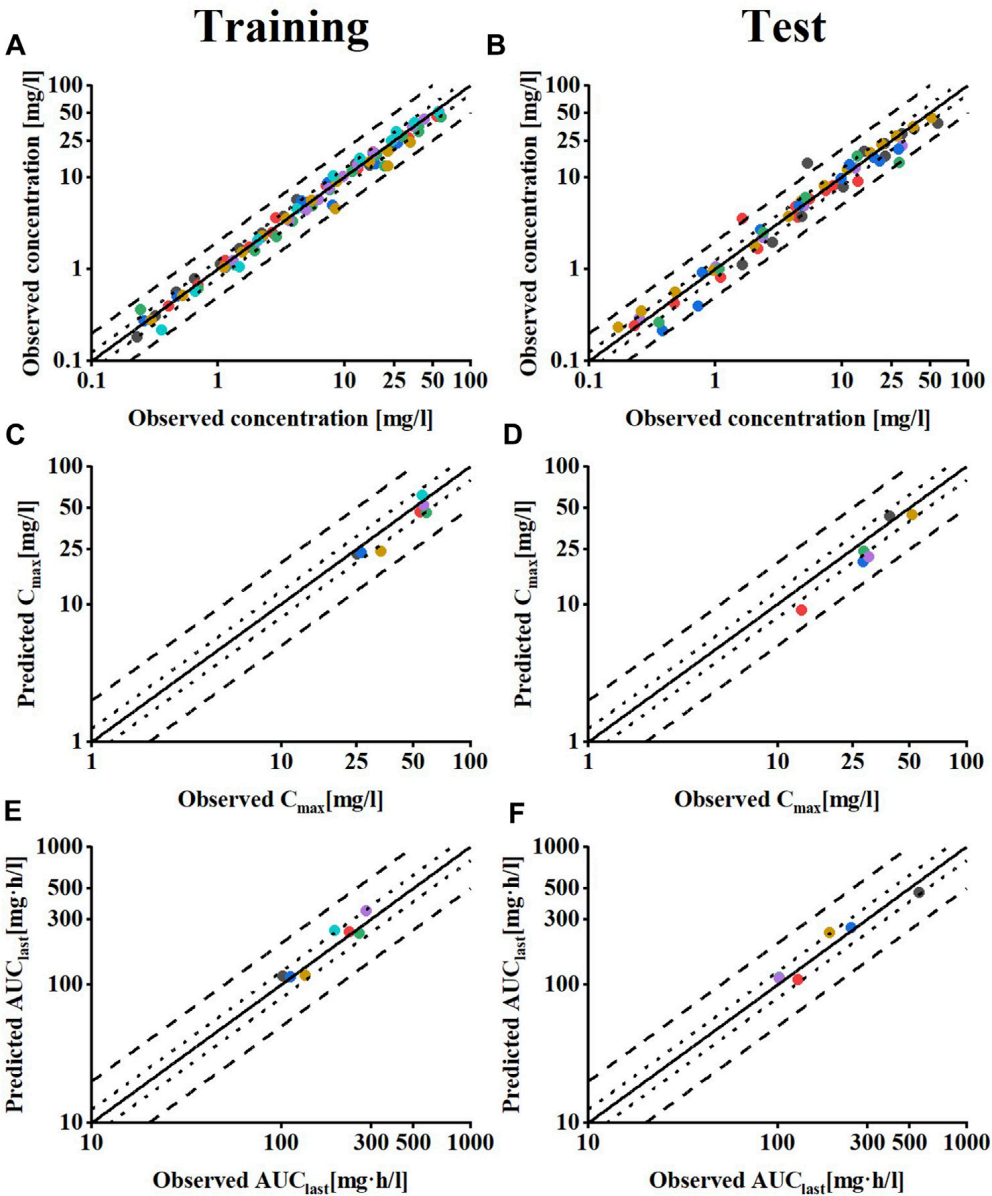
Model performance evaluation of meropenem in healthy individuals. Predicted compared to observed meropenem **(A,B)** plasma concentrations **(C,D)** C_max_ and **(E,F)** AUC_last_ values of all clinical studies used, separated by training and test dataset. The solid line marks the line of identity. Dotted lines indicate 1.25-fold, dashed lines indicate 2-fold deviation. Details on the MRD and GMFE values are listed in Supplementary Tables S3–S5.

Regarding the model evaluation, Figure 2 shows goodness-of-fit plots comparing all predicted *versus* observed concentration measurements and AUC_last_, as well as C_max_ values of meropenem, divided into training and test datasets. Overall, the predicted concentration measurements (98.7%), AUC_last_ (13/13), and C_max_ values (13/13) are mainly within the 2-fold range of their respective observed counterparts. The prediction model shows the overall MRD of 1.25 for predicted concentration measurements and GMFE values of 1.14 for the AUC_last_ and 1.26 for predicted C_max_ values, indicating a good model performance.

### 3.2 CKD PBPK model development

**FIGURE 3:**
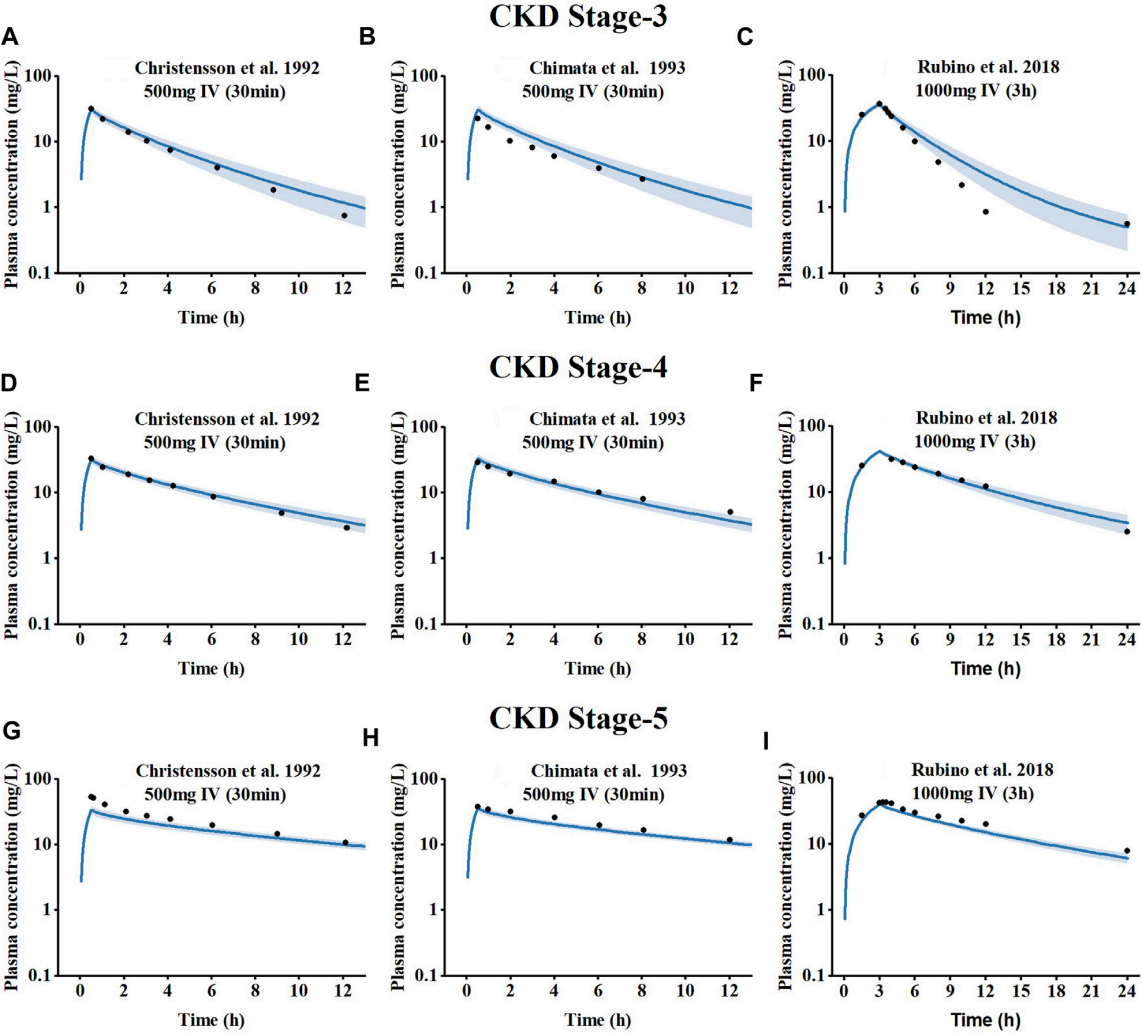
Simulated plasma concentration-time profiles for meropenem at different CKD stages, compared to observed data. **(A–C)** CKD stage 3, **(D–F)** CKD stage 4, and **(G–I)** CKD stage 5.The shaded area indicates the corresponding standard deviation (SD) for the simulated data, and the black circles represent the mean observed concentration for each respective study.

The results of the PBPK model scaling to CKD meropenem are depicted in Figure 3, compared with the measured concentration values obtained from clinical studies in patients at different stages of CKD. The visual predictive checks indicated that meropenem exposure increased with worsening CKD due to the fact that meropenem plasma concentrations declined more slowly with decreasing renal function. Goodness-of-fit plots were used to evaluate the model further, and the predicted pharmacokinetic parameters appeared to be within the two fold acceptance criterion, shown in Supplementary Figure S3 in the ESM. The overall MRD is 1.36, and the GMFE of AUC_last_ and C_max_ are 1.24 and 1.28, respectively. Detailed results on GMFE and MRD values calculated for all studies are given in Supplementary Tables S6–S8. These results indicated that the optimized PBPK model was able to precisely the meropenem exposure *in vivo* in patients at different stages of CKD.

### 3.3 Intermittent hemodialysis (IHD) PBPK model

**FIGURE 4:**
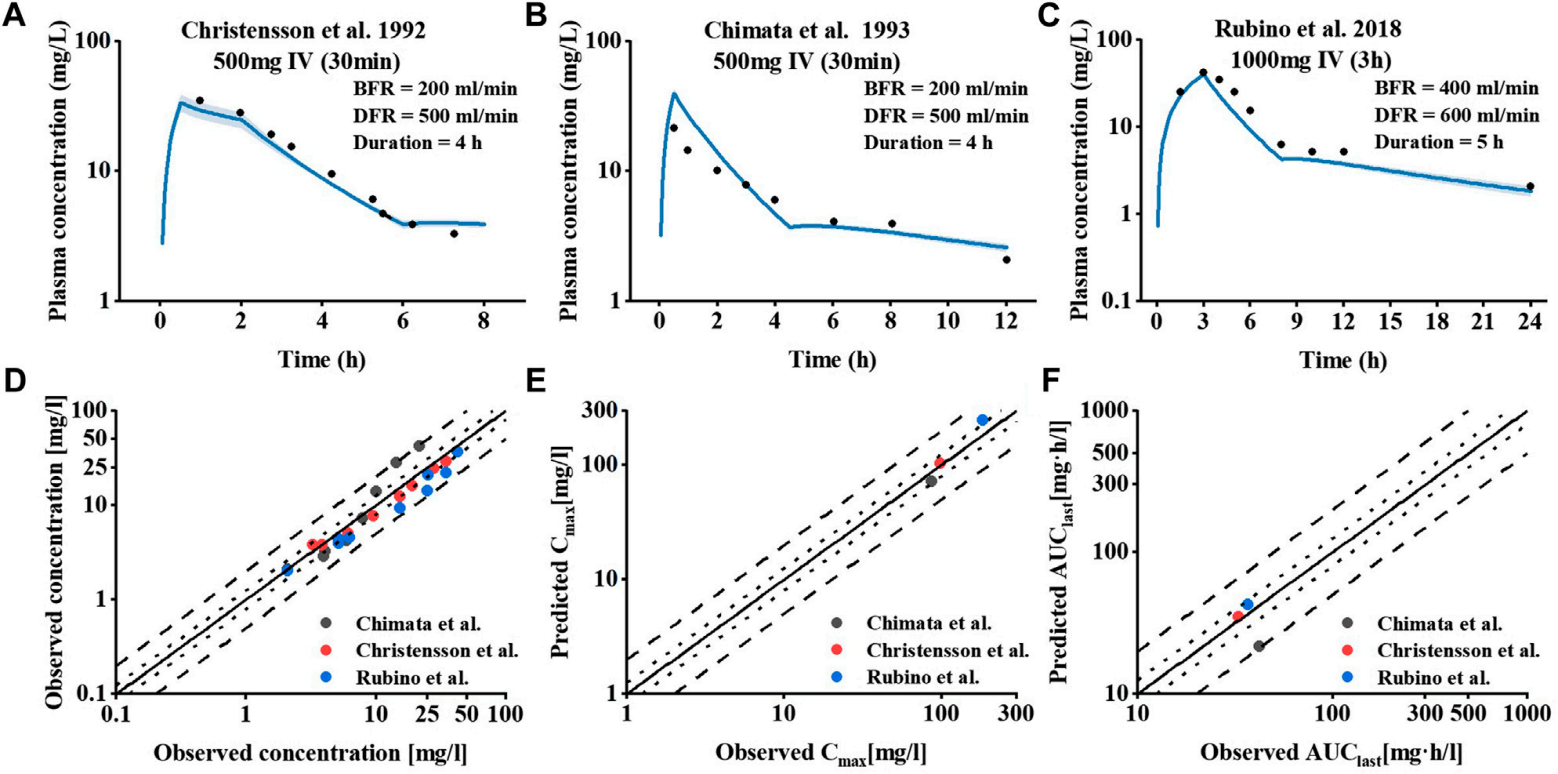
Meropenem plasma concentration and model performance in patients receiving IHD. **(A–C)** Simulated and observed plasma concentration–time profiles at different dialysis parameter settings. The shaded area indicates the corresponding standard deviation (SD) for the simulated data, and the black circles represent the mean observed concentration for each respective study. **(D–F)** Meropenem hemodialysis model performance.

To investigate the effect of hemodialysis on the plasma concentration of meropenem, an extension of the meropenem CKD model was developed by adding a dialyzer compartment in MoBi and then adapting the equation defined by Michaels to describe the clearance efficiency of the dialyzer (Michaels, 1966). Visual comparisons of predicted to observed concentration-time profiles of meropenem in dialysis patients are shown in Figures 4A–C. Meropenem declined more rapidly during the on-dialysis session than during the off-dialysis session. The predictions of plasma concentration-time trajectories in different hemodialysis parameter settings are in close agreement with observed plasma concentration data. Since Cmax was not accurately predicted. The curve trajectory appeared slightly deviated, as shown in Figure 4B. Moreover, goodness-of-fit plots of predicted to observed AUClast, Cmax and concentration measurements are shown in Figures 4D–F. In summary, 100% of the predicted parameter values were within two-fold of the observed values. The MRD value for meropenem IHD model was 1.36, and the GMFE values were 1.20 and 1.39 for the predicted AUClast and Cmax (presented in Supplementary Tables S6–S8 of the ESM).

### 3.4 Pharmacodynamic evaluation of CKD individuals

**FIGURE 5:**
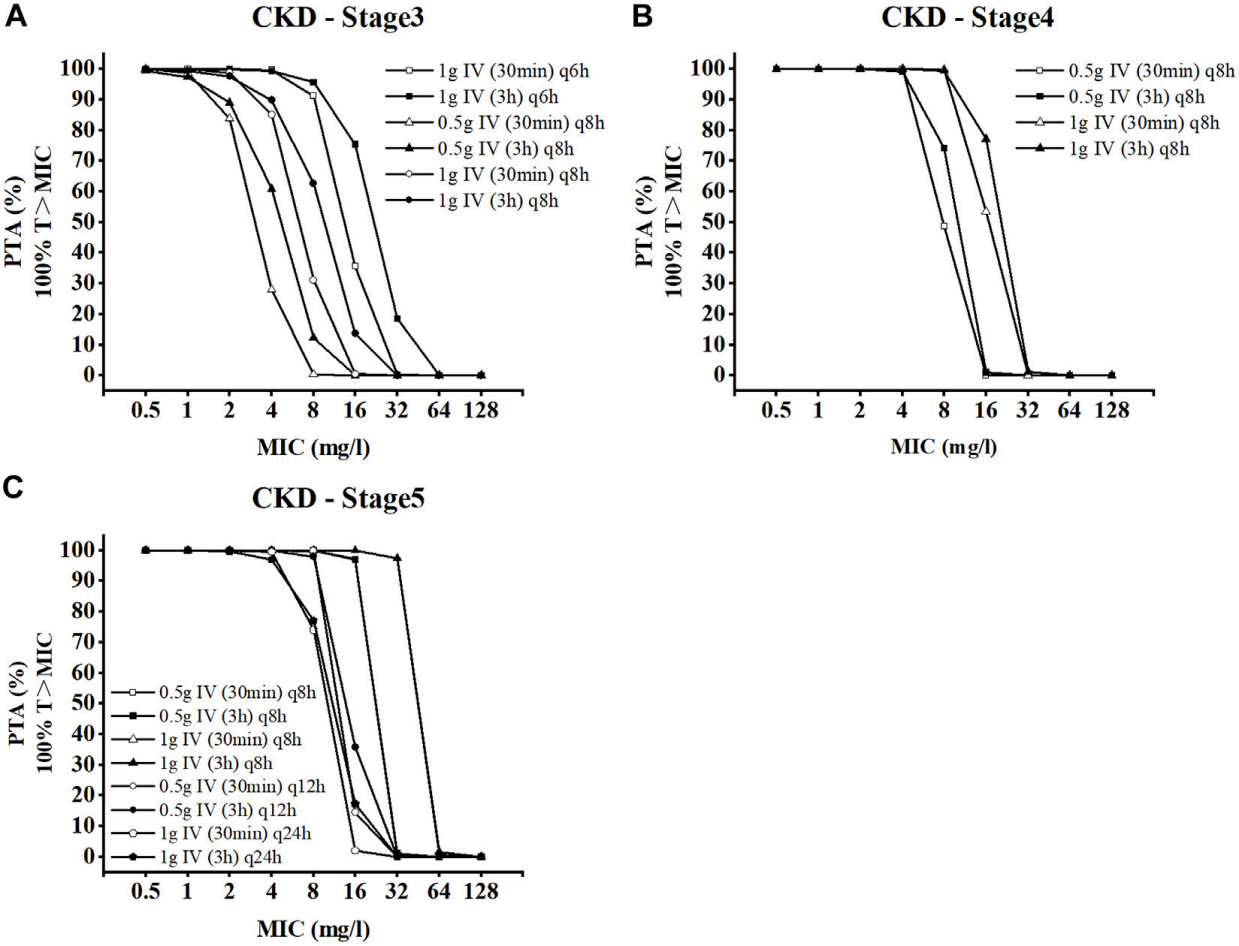
Probability of target attainment for CKD dose regimens of meropenem as 3-h and 30-min infusions. **(A)** CKD stage 3, **(B)** CKD stage 4, and **(C)** CKD stage 5.

An MCS with 20,000 subjects was performed to calculate PTA values based on the PK data for meropenem from the different regimens. The PTA values for the dosing regimens applied to the CKD virtual populations with different renal functions are shown in Figure 5. In comparison to the conventional dose infusion (30min), the PTA values of prolonged infusion (3 h) were, on average, greater. In the CKD-stage3 simulations, the dosing regimen with a total daily dose of 4 g (1 g iv q6h) had the best effect. The PTA of other dosing regimens failed to exceed 90% when the MIC was greater than 2 mg/L. With the gradual deterioration of renal function, the patient’s drug intake under the conventional dosing regimen is excessively high, which needs to be adjusted in accordance with the patient’s renal function. In CKD-stage5 simulations, pharmacodynamic goals were also successfully attained with the proper dose reduction (0.5 g iv q12 h or 1 g iv once day). When the MIC was 8 mg/L, the PTA of regimen 1 g iv once daily was reduced to less than 90%. For patients with CKD-stage 5, the dose regimen of 0.5 g iv every 12 h is preferable to the dose regimen of 1 g iv once daily.

### 3.5 Pharmacodynamic evaluation of IHD individuals

We simulated the achievement of the pharmacodynamic target within 1 week for IHD patients under various dosing regimens as part of the pharmacodynamic evaluation of the dialysis model, and the pharmacodynamic target was established at 40% T > MIC. The simulated hemodialysis population adopted a three times per week schedule. The dialysis duration of the simulation was set to 4h, the BFR was set to 200 mL/min, and the DFR was set to 500 mL/min. Table 3 shows the PTA on the day of dialysis and between dialysis sessions for a week. When the MIC was less than or equal to 4 mg/L, PTAs for all dosing regimens exceeded 90% at a 40%T > MIC on the off-dialysis session. During dialysis, the probability of a partial dosing regimen is reduced. When the MIC is greater than or equal to 2 mg/L, the PTA of 0.25 g iv once daily regimen is less than 90%. When a MIC was 4 mg/L, the PTA of the 0.5 g iv once daily dosing regimen was close to 90, but not reached. When a MIC was 8 mg/L, a dosing regimen of 0.5 g iv q12 h is optimal.

**TABLE 3:** PTA(%) at 40%T > MIC during dialysis or non-dialysis in 1 week in patients receiving intermittent hemodialysis.

Meropenem IV regimen	Dialysis session	MIC (mg/L)			
		1	2	4	8
0.25 g once daily	on-dialysis	100	84.0	2.09	0
	off-dialysis	100	100	99.2	0.34
0.25 g q12h	on-dialysis	100	100	100	85.6
	off-dialysis	100	100	100	99.6
0.5 g once daily	on-dialysis	100	100	84.6	2.26
	off-dialysis	100	100	100	99.2
0.5 g q12h	on-dialysis	100	100	100	100
	off-dialysis	100	100	100	100
1 g once daily	on-dialysis	100	100	100	85.8
	off-dialysis	100	100	100	100

## 4 Discussion

At present, there are few reports on the pharmacokinetics of meropenem in CKD and hemodialysis patients, and there is a lack of reference medication information. This makes a rational drug use in special populations a significant challenge. PBPK models support model-based drug delivery when clinical data are lacking, but the application of these methods in patients with impaired renal function is not fully mature. This study aims to develop a new PBPK modeling framework for predicting the pharmacokinetic changes of meropenem in CKD and hemodialysis patients and provide a dose adjustment scheme, which provides the possibility to use PBPK models to guide meropenem medication regimens in the future.

In this study, a whole-body PBPK model of meropenem for healthy individuals was developed and then scaled to the CKD population. In the process of CKD-PBPK model construction, we adjusted the concentrations levels of OAT3 and NPT1, two transporters that mediated the renal tubular secretion of meropenem, according to the INH hypothesis. We assumed that the decrease in the concentrations of these two transporters was synchronized. Studies have shown that renal injury may lead to the downregulation of OATs expression levels (Wang and Sweet, 2013), while the performance of NPT1 in CKD is still unclear. Nevertheless, the established CKD model could accurately predict the in plasma exposure of meropenem in patients with different CKD stages. In order to further verify the reliability of the model, different administration schemes (500 mg IV 30 min or 1,000 mg IV 3 h) were included in the CKD model, compared with the observed value of the corresponding clinical data. Of note, the model performed well, with predictions meeting the two-fold error threshold criterion (Supplementary Figure S3). Our results showed that meropenem clearance efficiency decreases with decreased renal function, leading to the result of increased plasma exposures of meropenem (Figure 3). Sensitivity analysis showed that renal volume had a significant effect on the AUC of meropenem (Supplementary Figures S4–S6). With the deterioration of CKD, the sensitivity of OAT3 and NPT1 related dynamic parameters decreased, as kidney volume and renal perfusion are declined.

Then, we extended the CKD model by adding a dialyzer compartment to the ESRD PBPK model to establish an IHD PBPK model and successfully predicted the *in vivo* pharmacokinetics of meropenem induced by hemodialysis. There are still few PBPK models of dialysis, extracorporeal membrane oxygenation (ECMO). Watt et al. (2018) developed a PBPK model of fluconazole in children on ECMO and used it to predict the optimal dose for ECMO-treated children of all ages. Fuhr et al. (2020) established an idarucizumab-dabigatran-hemodialysis PK/PD model to study and predict outcomes of different dabigatran reversal regimens and to develop individualized treatment regimens for patients with reduced renal function. Studies on drug administration regimens for these patients are primarily in clinical pharmacokinetic studies, and studies on other aspects are still lacking. To our knowledge, this study is the first PBPK modeling method for meropenem in a IHD population.

After the construction of CKD and hemodialysis PBPK models, we combined Monte Carlo method for pharmacodynamic evaluation, simulated and evaluated the dosing regimen for CKD and hemodialysis virtual populations. The MIC sensitivity breakpoint of meropenem to the most prevalent bacteria is less than 8 mg/L. For example, the MIC sensitivity breakpoint of Enterobacteriaceae is ≤0.25 mg/L, *Acinetobacter* baumannii and *P. aeruginosa* is ≤2 mg/L. When MIC is greater than 8 mg/L carbapenems are no longer recommended for the treatment of CRE (carbapenem-resistant *E. coli*) infection. Our results suggested that the dosing strategies with prolonged infusion time help to improve PTA in the early stages of CKD. Dose adjustments are recommended with worsening renal function. Appropriate dose reductions are required to accommodate the reduced renal function in CKD patients. According to the simulation results (Table 3), all the simulated dosing regimens for a MIC ≤8 mg/L were met with the pharmacodynamic target between the hemodialysis sessions, except for 0.25 g iv once daily. Among them, we consider a dosage of 0.5 g iv once daily as the optimal regimen for IHD patients the interdialysis period. On the day of dialysis, completion of the meropenem infusion before the initiation of dialysis may substantially reduce exposure leading to poor PTA results. When MIC was 8 mg/L, only a dosage of 0.5 g iv q12 h reached PTA greater than 90% during dialysis. The PTA of the 1 g iv once daily dosing regimen was close to 90, but not reached. There is currently inadequate information regarding the use of meropenem in patients on hemodialysis. Czock and Keller (2014) recommended 1 g once daily as the dosing regimen for IHD patients on the day of dialysis. Yokoyama et al. (2018) suggested 0.5 g once daily as an appropriate regimen for empirical treatment. Since the exposure of meropenem in hemodialysis patients is affected by the setting of hemodialysis parameters (Supplementary Figure S7), dose adjustments are recommended according to the actual situation in clinical practice.

Our study still has some limitations. Firstly, many of the parameters in the model were fitted or assumed, such as Vmax of NPT1 and Km. Despite the fact that there is proof that npt1 is an efflux transporter that mediates the tubular secretion of meropenem (Uchino et al., 2000), details on the kinetic parameters of NPT1 with meropenem as the substrate are still absent. Moreover, the applicability of the INH model remains controversial. Huang and Isoherranen (2020) believed that the INH model is not suitable for drugs with medium and high permeability and is only suitable for drugs with low permeability and non-reabsorption. Meropenem is a highly renally secreted drug, whereas reabsorption has less of an impact on the contribution of meropenem overall excretion. Although our results showed that the meropenem CKD-PBPK model was successfully constructed based on the INH hypothesis, whether the INH model used in this study could be applied to drugs with different renal processing pathways, such as highly reabsorbed drugs, remains to be determined. Therefore, in the future, the model can be further refined to include drugs with different renal elimination to test the applicability of the INH model.

## 5 Conclusion

In this study, the established PBPK model successfully predicted the plasma concentration distribution of meropenem in healthy individuals, CKD and IHD populations. Furthermore, the PBPK model was then used to evaluate dosing regimens in CKD and IHD populations at different MIC values by MCS. MCS results showed that 0.5 g iv once daily was the optimal dosing regimen for the interdialysis period, while a larger dose of 0.5 g iv q12 h was required on the day of dialysis when MIC ≤8 mg/L and the pharmacodynamic target was 40% *f* T > MIC. The final model may serve as a useful tool to further study the effects of CKD and hemodialysis on drug pharmacokinetics, and to facilitate dosing regimen decision-making in CKD and IHD populations.

## Data Availability

The original contributions presented in the study are included in the article/Supplementary Material, further inquiries can be directed to the corresponding authors.
