# Supplementary material for: Physiologically based pharmacokinetic-pharmacodynamic evaluation of meropenem in CKD and hemodialysis individuals
Source: Front Pharmacol. 2023 Mar 7;14:1126714. doi: 10.3389/fphar.2023.1126714 (PMC10027930; doi:10.3389/fphar.2023.1126714)
Supplement: Supplementary file 1 [file DataSheet1.docx]

Supplementary Material for

Physiologically based pharmacokinetic-pharmacodynamic evaluation of meropenem in CKD and hemodialysis individuals

Guoliang Deng^1,2^, Fan Yang^3^, Ning Sun^4^, Danhong Liang^1,2^, Anfen Cen^1,2^, Chen Zhang^1,2*^ and Suiqin Ni^1,2*^

^1^School of Biology and Biological Engineering, South China University of Technology, Guangzhou, Guangdong, China

^2^Department of Pharmacy, the Second Affiliated Hospital, School of Medicine, South China University of Technology, Guangzhou, Guangdong, China

^3^Department of Hepatobiliary Surgery, Guangzhou Eighth People's Hospital, No. 8 Huaying Road, Baiyun District, Guangzhou, Guangdong, China

^4^Guangzhou First People's Hospital, School of Medicine, South China University of Technology, Guangzhou, Guangdong, China.

*** Correspondence:**Chen Zhang, [eyzhangchen@scut.edu.cn](mailto:eyzhangchen@scut.edu.cn); Suiqin Ni, [ywjd510@126.com](mailto:ywjd510@126.com)

Supplementary Table S1: Clinical study data of meropenem in healthy individuals

| Dose [mg] | Infusion  time | n | Female [%] | Age(SD) [years] | Weight(SD) [kg] | Height(SD) [cm] | C_max_  [mg/l] | AUC_0-inf_  [mg·h/l] | Population | Dataset | References |
| --- | --- | --- | --- | --- | --- | --- | --- | --- | --- | --- | --- |
| 500 | 30 min | 6 | 0 | 26 [19-45] | 74 [68-87] | 179 [170-184] | 25.6 (2.5) | 30.1 (2.1) | Healthy | Training | Bax et al. (1989) |
| 500 | 30 min | 5 | 25 | 45 (2) | 70.4 (1.2) | 174 (2) | 26.9 (1.7) | 32.4 (1.4) | Healthy | Training | Harrison et al. (1993) |
| 500 | 30 min | 8 | 0 | 28 (5.2) | 68.6 (7.7) | - | 35.6 (5.2) | 39.6 (6.8) | Healthy | Training | Ljungberg and Nilsson-Ehle (1992) |
| 1000 | 5 min | 6 | 0 | 23.6 [23-31] | 69.9 [63-80] | 180 [169-187] | 55.8 (9.4) | 66.9 (13.7) | Healthy | Training | Wise et al. (1990) |
| 1000 | 30 min | 6 | 0 | 26 [19-45] | 74 [68-87] | 179 [170-184] | 55.4 ( 3.7) | 66.9 (4.6) | Healthy | Training | Bax et al. (1989) |
| 1000 | 30 min | 8 | 0 | 33 [22-38] | 74 [66-86] | - | 61.6 (6.8) | 77.5 (11.5) | Healthy | Training | Nilsson-Ehle et al. (1991) |
| 500 | 30 min | 6 | - | 33.8 (9) | 66.9 (13.6) | - | 28.6 (11.9) | 36.0 (17.6) | Healthy | Test | Leroy et al. (1992) |
| 500 | 30 min | 6 | 0 | 34 (13.4) | 79 (8.4) | - | 30.3 (3.6) | 36.0 (4.5) | Healthy | Test | Christensson et al. (1992) |
| 500 | 180 min | 6 | 0 | 34 (12) | 82.1 (18.5) | - | 9.71 (0.76) | 36.0 (2.9) | Healthy | Test | Dandekar et al. (2003) |
| 1000 | 30 min | 12 | 0 | 29.4 (6) | 80.3 (7.2) | - | 51.6 (6.5) | 70.5 (10.3) | Healthy | Test | Dreetz et al. (1996) |
| 1000 | 180 min | 8 | 50 | 56.8 (3.4) | 91.6 (11.0) | 169.6 (9.1) | 27.5 (7.03) | 87.1 (26.7) | Healthy | Test | Rubino et al. (2018b) |
| 2000 | 180 min | 6 | 0 | 34 (12) | 82.1 (18.5) | - | 39.76 (6.39) | 159.6 (28.8) | Healthy | Test | Dandekar et al. (2003) |

“[]”Square brackets represent the range of values ; Cmax, maximum observed plasma concentration; AUC_0–inf_, area under the plasma concentration-time curve from time zero to infinity;

Supplementary Table S2: Clinical study data of meropenem in individuals at different stages of CKD

| Dose [mg] | Infusion time | n | Female [%] | Age(SD) [years] | Weight(SD) [kg] | Height(SD) [cm] | C_max_  [mg/l] | AUC_0-inf_  [mg·h/l] | Population | Dataset | References |
| --- | --- | --- | --- | --- | --- | --- | --- | --- | --- | --- | --- |
| 500 | 30 min | 4 | 25 | 54 (6) | 64.8 (14.9) | - | 22.5 | 74.6 (29.0) | CKD-stage3 | Test | Chimata et al. (1993) |
| 500 | 30 min | 5 | 60 | 64.6 (10.8) | 54 (3.3) | - | 28.9 | 186.8 (68.5) | CKD-stage4 | Test | Chimata et al. (1993) |
| 500 | 30 min | 4 | 100 | 54.5 (8.65) | 45 (10.3) | - | 37.9 | 392 | CKD-stage5 | Test | Chimata et al. (1993) |
| 500 | 30 min | 4 | 50 | 56.5 (12.7) | 48 (8) | - | 21.5 | 86.6 | ESRD on hemodialysis | Test | Chimata et al. (1993) |
| 500 | 30 min | 5 | 0 | 47 (19.1) | 84 (9.3) | - | 31.7 (5.1) | 89.8 (17.9) | CKD - Stage3 | Test | Christensson et al. (1992) |
| 500 | 30 min | 7 | 28.8 | 53 (11.2) | 76 (12.4) | - | 33.1 (5.8) | 156 (63.8) | CKD - Stage4 | Test | Christensson et al. (1992) |
| 500 | 30 min | 5 | 60 | 37 (5.3) | 67 (18.7) | - | 53.1 (10.9) | 393 (83.8) | CKD - Stage5 | Test | Christensson et al. (1992) |
| 500 | 30 min | 5 | 60 | 37 (5.3) | 67 (18.7) | - | 34.8 | 111.9 | ESRD on hemodialysis | Test | Christensson et al. (1992) |
| 1000 | 180 min | 8 | 50 | 62.1 (10.2) | 89.2 (21.4) | 170.9 (8.1) | 40.5 (6.27) | 181 (59.4) | CKD-stage3 | Test | Rubino et al. (2018a) |
| 1000 | 180 min | 8 | 37.5 | 57.6 (6.4) | 94.2 (19.4) | 174.4 (9.6) | 45.5 (13.0) | 397 (98.0) | CKD-stage4 | Test | Rubino et al. (2018a) |
| 1000 | 180 min | 9 | 0 | 53.8 (10.5) | 106.0 (12.9) | 177.8 (4.6) | 47.7 (11.7) | 629 (206) | CKD-stage5 | Test | Rubino et al. (2018a) |
| 1000 | 180 min | 9 | 0 | 53.8 (10.5) | 106.0 (12.9) | 177.8 (4.6) | 44.7 (8.40) | 280 (58.7) | ESRD on hemodialysis | Test | Rubino et al. (2018a) |

ESRD: end-stage renal disease; Cmax, maximum observed plasma concentration; AUC_0–inf_, area under the plasma concentration-time curve from time zero to infinity;


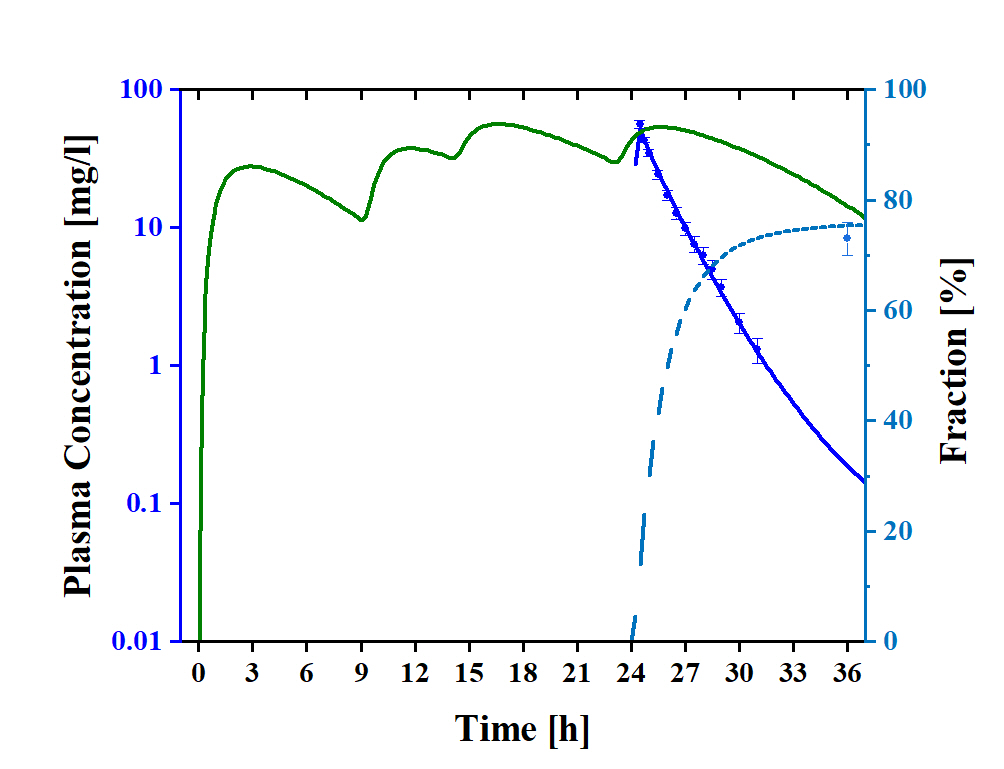


Figure S1 The simulated and observed plasma concentration-time profiles of meropenem (1g) with probenecid (0.5g) in healthy individuals. Clinical observed data (Bax et al., 1989) are shown as mean ± SD (circles). Solid lines illustrate the predicted plasma concentrations, probenecid in green and meropenem in dark blue. Dashed light blue lines represent the predicted fractions excreted to urine.

Supplementary Table S3: Mean relative deviation (MRD) values of the predicted meropenem plasma concentrations for healthy individuals

| Reference | Dose [mg] | Infusion time | Dataset | MRD |
| --- | --- | --- | --- | --- |
| Bax et al. (1989) | 500 | 30min | Training | 1.16 |
| Harrison et al. (1993) | 500 | 30min | Training | 1.21 |
| Ljungberg and Nilsson-Ehle (1992) | 500 | 30min | Training | 1.29 |
| Wise et al. (1990) | 1000 | 5min | Training | 1.24 |
| Bax et al. (1989) | 1000 | 30min | Training | 1.11 |
| Bax et al. (1989) | 1000 | 30min | Training | 1.07 |
| Nilsson-Ehle et al. (1991) | 1000 | 30min | Training | 1.24 |
| Christensson et al. (1992) | 500 | 30min | Test | 1.19 |
| Leroy et al. (1992) | 500 | 30min | Test | 1.39 |
| Dreetz et al. (1996) | 1000 | 30min | Test | 1.16 |
| Dandekar et al. (2003) | 500 | 3h | Test | 1.33 |
| Rubino et al. (2018b) | 1000 | 3h | Test | 1.38 |
| Dandekar et al. (2003) | 2000 | 3h | Test | 1.45 |
|  |  |  | MRD (range) | 1.25 (1.07-1.45) |
|  |  |  | MRD < 2 | 13/13 subjects |

Supplementary Table S4: Observed and predicted AUC values of meropenem for healthy individuals with geometric mean fold errors (GMFE)

| Reference | Dose [mg] | Infusion time | Dataset | AUC_pred_ [mg·h/l] | | AUC_obs_ [mg·h/l] | AUC_pred_/AUC_obs_ |
| --- | --- | --- | --- | --- | --- | --- | --- |
| Bax et al. (1989) | 500 | 30min | Training | 115.18 | | 101.92 | 1.13 |
| Harrison et al. (1993) | 500 | 30min | Training | 115.05 | | 112.64 | 1.02 |
| Ljungberg and Nilsson-Ehle (1992) | 500 | 30min | Training | 116.76 | | 134.74 | 0.87 |
| Wise et al. (1990) | 1000 | 5min | Training | 248.31 | | 191.22 | 1.30 |
| Bax et al. (1989) | 1000 | 30min | Training | 240.66 | | 230.41 | 1.04 |
| Bax et al. (1989) | 1000 | 30min | Training | 342.31 | | 281.12 | 1.22 |
| Nilsson-Ehle et al. (1991) | 1000 | 30min | Training | 235.23 | | 259.43 | 0.91 |
| Christensson et al. (1992) | 500 | 30min | Test | 111.57 | | 102.49 | 1.09 |
| Leroy et al. (1992) | 500 | 30min | Test | 113.41 | | 102.34 | 1.11 |
| Dreetz et al. (1996) | 1000 | 30min | Test | 238.22 | | 189.21 | 1.26 |
| Dandekar et al. (2003) | 500 | 3h | Test | 109.14 | | 128.53 | 0.85 |
| Rubino et al. (2018b) | 1000 | 3h | Test | 258.16 | | 245.75 | 1.05 |
| Dandekar et al. (2003) | 2000 | 3h | Test | 468.46 | | 562.83 | 0.83 |
|  |  |  |  |  | GMFE (range) | | 1.14 (1.02-1.26) |
|  |  |  |  |  | GMFE < 2 | | 13/13 subjects |

AUC: area under the plasma concentration-time curve

Supplementary Table S5: Observed and predicted C_max_ values of meropenem for healthy individuals with GMFE

| Reference | Dose [mg] | Infusion time | Dataset | Pred C_max_ [mg/l] | | Obs C_max_ [mg/l] | (Pred C_max_)/(Obs C_max_ ) | |  |
| --- | --- | --- | --- | --- | --- | --- | --- | --- | --- |
| Bax et al. (1989) | 500 | 30min | Training | 23.03 | | 25.21 | | 0.91 | |
| Harrison et al. (1993) | 500 | 30min | Training | 23.69 | | 26.43 | | 0.90 | |
| Ljungberg and Nilsson-Ehle (1992) | 500 | 30min | Training | 24.08 | | 33.56 | | 0.72 | |
| Wise et al. (1990) | 1000 | 5min | Training | 61.54 | | 55.80 | | 1.10 | |
| Bax et al. (1989) | 1000 | 30min | Training | 46.62 | | 54.19 | | 0.86 | |
| Bax et al. (1989) | 1000 | 30min | Training | 52.11 | | 56.44 | | 0.92 | |
| Nilsson-Ehle et al. (1991) | 1000 | 30min | Training | 46.24 | | 58.78 | | 0.79 | |
| Christensson et al. (1992) | 500 | 30min | Test | 22.07 | | 30.30 | | 0.73 | |
| Leroy et al. (1992) | 500 | 30min | Test | 24.06 | | 28.61 | | 0.84 | |
| Dreetz et al. (1996) | 1000 | 30min | Test | 44.60 | | 51.60 | | 0.86 | |
| Dandekar et al. (2003) | 500 | 3h | Test | 9.04 | | 13.47 | | 0.67 | |
| Rubino et al. (2018b) | 1000 | 3h | Test | 20.19 | | 28.29 | | 0.71 | |
| Dandekar et al. (2003) | 2000 | 3h | Test | 43.49 | | 39.24 | | 1.11 | |
|  |  |  |  | | GMFE (range) | | 1.26 (1.08-1.50) | |  |
|  |  |  |  | | GMFE < 2 | | 13/13 subjects | |  |


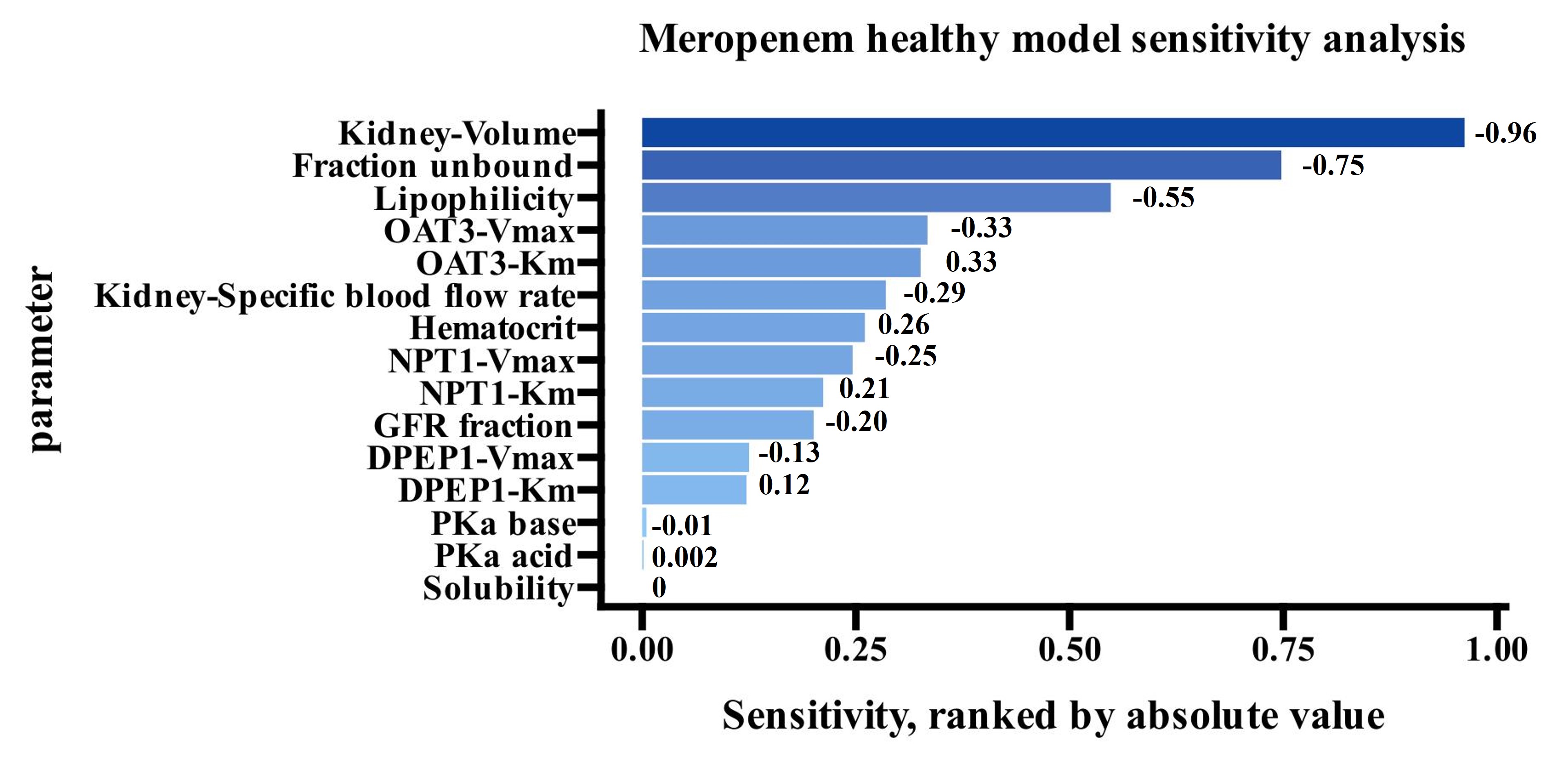


Supplementary Figure S2: Meropenem healthy PBPK model sensitivity analysis. Sensitivity to single parameters, measured as change of the simulated AUC_last_ of a 500 mg intravenous infusion for 30min. Km, Michaelis–Menten constant; Vmax, maximum rate of reaction.

Supplementary Table S6: MRD values of the predicted meropenem plasma concentrations for individuals at different stages of CKD as well as hemodialysis individuals

| Reference | State | Dose [mg] | | Infusion time | MRD |  |
| --- | --- | --- | --- | --- | --- | --- |
| Christensson et al. (1992) | CKD - Stage3 | 500 | | 30min | 1.44 |  |
| Christensson et al. (1992) | CKD - Stage4 | 500 | | 30min | 1.46 |  |
| Christensson et al. (1992) | CKD - Stage5 | 500 | | 30min | 1.40 |  |
| Christensson et al. (1992) | ESRD with hemodialysis | 500 | | 30min | 1.19 |  |
| Chimata et al. (1993) | CKD - Stage3 | 500 | | 30min | 1.61 |  |
| Chimata et al. (1993) | CKD - Stage4 | 500 | | 30min | 1.34 |  |
| Chimata et al. (1993) | CKD - Stage5 | 500 | | 30min | 1.11 |  |
| Chimata et al. (1993) | ESRD with hemodialysis | 500 | | 30min | 1.50 |  |
| Rubino et al. (2018a) | CKD - Stage3 | 1000 | | 3h | 1.44 |  |
| Rubino et al. (2018a) | CKD - Stage4 | 1000 | | 3h | 1.13 |  |
| Rubino et al. (2018a) | CKD - Stage5 | 1000 | | 3h | 1.28 |  |
| Rubino et al. (2018a) | ESRD with hemodialysis | 1000 | | 3h | 1.41 |  |
|  |  |  | MRD (range) | | 1.36 (1.11-1.61) | |
|  |  |  | MRD < 2 | | 12/12 subjects | |

Supplementary Table S7: Observed and predicted AUC_last_ values of meropenem for CKD and hemodialysis individuals with GMFE

| Reference | State | Dose [mg] | Infusion time | AUCpred [mg·h/l] | | AUCobs [mg·h/l] | AUCpred/AUCobs |
| --- | --- | --- | --- | --- | --- | --- | --- |
| Christensson et al. (1992) | CKD - Stage3 | 500 | 30min | 343.35 | | 285.01 | 1.20 |
| Christensson et al. (1992) | CKD - Stage4 | 500 | 30min | 618.21 | | 521.32 | 1.19 |
| Christensson et al. (1992) | CKD - Stage5 | 500 | 30min | 1058.99 | | 1302.14 | 0.81 |
| Christensson et al. (1992) | ESRD with hemodialysis | 500 | 30min | 98.65 | | 103.89 | 0.95 |
| Chimata et al. (1993) | CKD - Stage3 | 500 | 30min | 368.79 | | 203.09 | 1.82 |
| Chimata et al. (1993) | CKD - Stage4 | 500 | 30min | 699.30 | | 503.17 | 1.39 |
| Chimata et al. (1993) | CKD - Stage5 | 500 | 30min | 926.62 | | 893.12 | 1.04 |
| Chimata et al. (1993) | ESRD with hemodialysis | 500 | 30min | 86.76 | | 72.54 | 1.20 |
| Rubino et al. (2018a) | CKD - Stage3 | 1000 | 3h | 522.21 | | 517.98 | 1.01 |
| Rubino et al. (2018a) | CKD - Stage4 | 1000 | 3h | 1138.86 | | 1149.59 | 0.99 |
| Rubino et al. (2018a) | CKD - Stage5 | 1000 | 3h | 1359.34 | | 1727.46 | 0.79 |
| Rubino et al. (2018a) | ESRD with hemodialysis | 1000 | 3h | 182.90 | | 246.18 | 0.74 |
|  |  |  |  | | GMFE (range) | | 1.23 (1.01-1.35) |
|  |  |  |  | | GMFE < 2 | | 12/12 subjects |

Supplementary Table S8: Observed and predicted C_max_ values of meropenem for CKD and hemodialysis individuals with GMFE

| Reference | State | Dose [mg] | Infusion time | Pred C_max_ [mg/l] | | Obs C_max_ [mg/l] | Pred C_max_/obs C_max_ |
| --- | --- | --- | --- | --- | --- | --- | --- |
| Christensson et al. (1992) | CKD - Stage3 | 500 | 30min | 26.76 | | 31.70 | 0.84 |
| Christensson et al. (1992) | CKD - Stage4 | 500 | 30min | 29.44 | | 33.10 | 0.89 |
| Christensson et al. (1992) | CKD - Stage5 | 500 | 30min | 32.97 | | 53.10 | 0.62 |
| Christensson et al. (1992) | ESRD with hemodialysis | 500 | 30min | 32.97 | | 34.84 | 0.95 |
| Chimata et al. (1993) | CKD - Stage3 | 500 | 30min | 32.34 | | 22.54 | 1.43 |
| Chimata et al. (1993) | CKD - Stage4 | 500 | 30min | 38.66 | | 28.91 | 1.34 |
| Chimata et al. (1993) | CKD - Stage5 | 500 | 30min | 46.20 | | 37.90 | 1.22 |
| Chimata et al. (1993) | ESRD with hemodialysis | 500 | 30min | 42.26 | | 21.52 | 1.96 |
| Rubino et al. (2018a) | CKD - Stage3 | 1000 | 3h | 30.40 | | 37.35 | 0.81 |
| Rubino et al. (2018a) | CKD - Stage4 | 1000 | 3h | 38.02 | | 31.67 | 1.20 |
| Rubino et al. (2018a) | CKD - Stage5 | 1000 | 3h | 36.88 | | 43.49 | 0.85 |
| Rubino et al. (2018a) | ESRD with hemodialysis | 1000 | 3h | 36.88 | | 42.67 | 0.86 |
|  |  |  |  | | GMFE (range) | | 1.3 (1.06-1.96) |
|  |  |  |  | | GMFE < 2 | | 12/12 subjects |


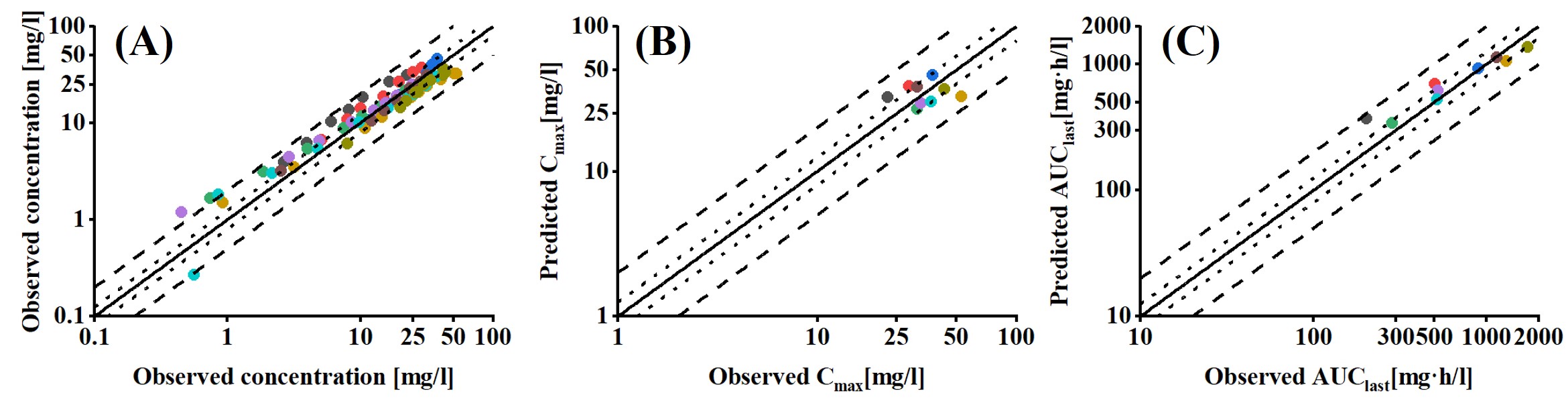


Supplementary Figure S3: Meropenem CKD PBPK model performance. Predicted compared to observed meropenem (A) plasma concentrations, (B) C_max_ and (C) AUC_last_ values of all clinical studies used. The solid line marks the line of identity. Dotted lines indicate 1.25-fold, dashed lines indicate 2-fold deviation.


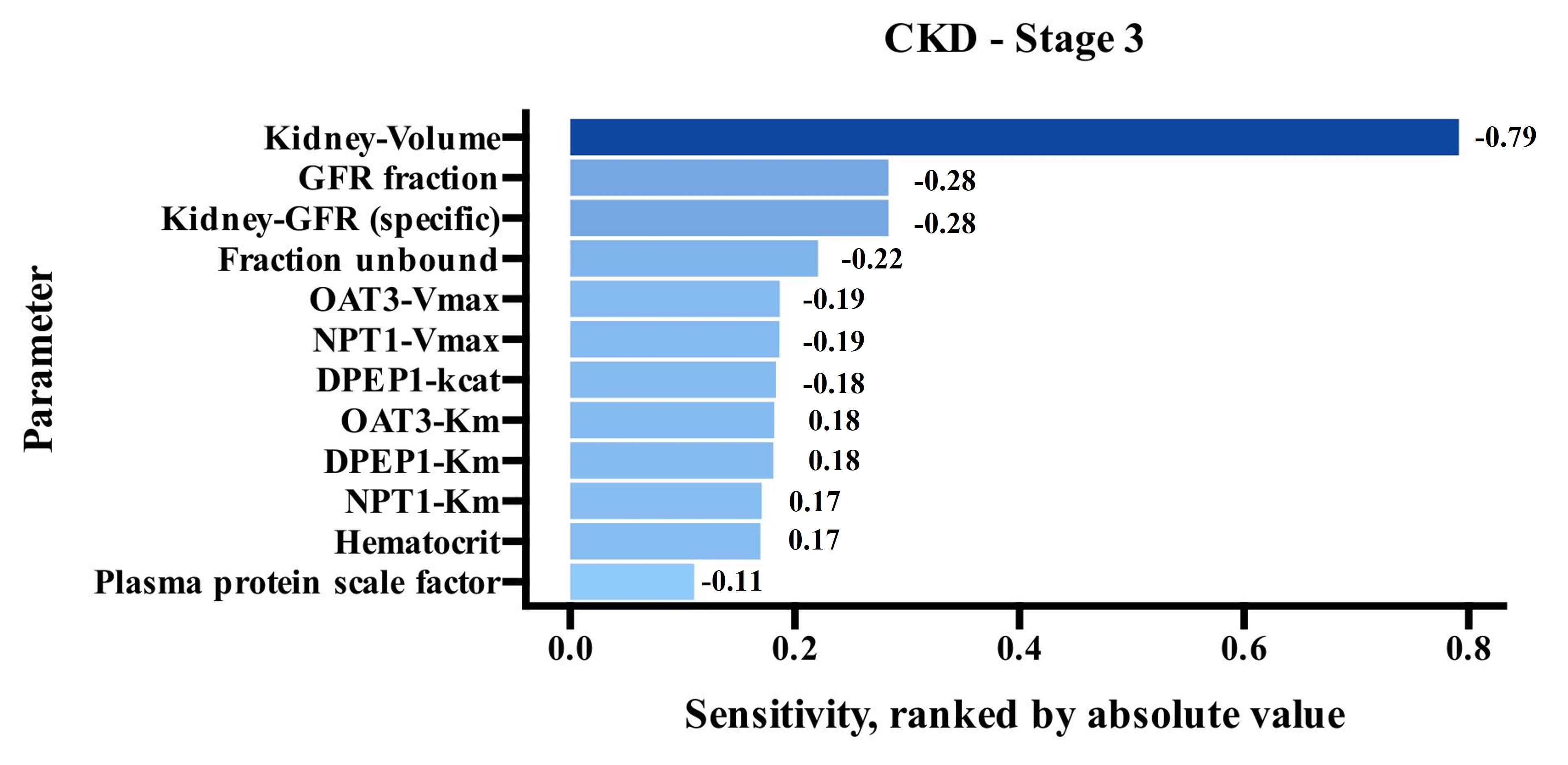


Supplementary Figure S4: Meropenem PBPK model sensitivity analysis in CKD stage 3. Sensitivity to single parameters, measured as change of the simulated AUC_last_ of a 500 mg intravenous infusion for 30min. Km, Michaelis–Menten constant; Vmax, maximum rate of reaction.


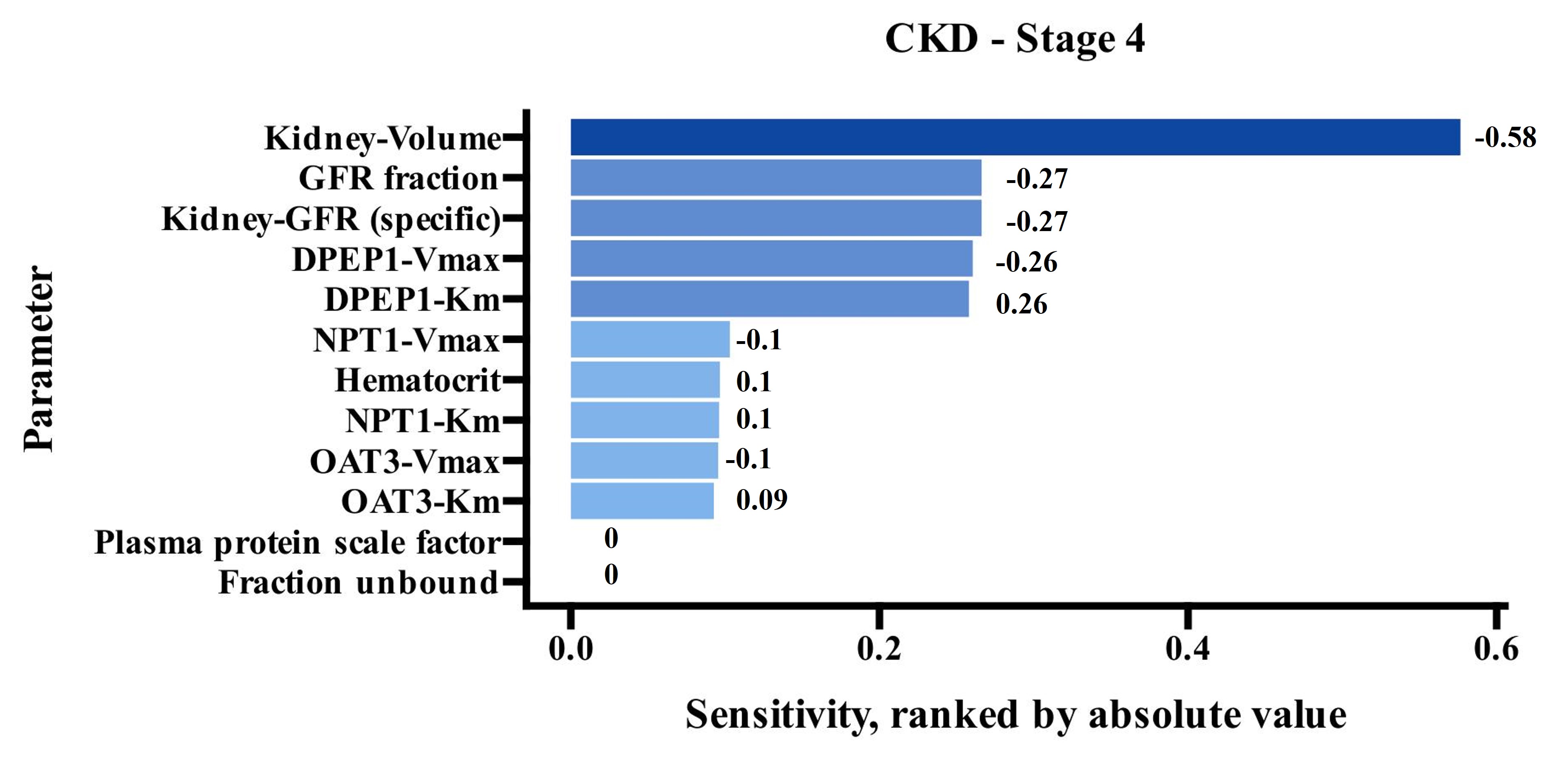


Supplementary Figure S5: Meropenem PBPK model sensitivity analysis in CKD stage 4. Sensitivity to single parameters, measured as change of the simulated AUC_last_ of a 500 mg intravenous infusion for 30min. Km, Michaelis–Menten constant; Vmax, maximum rate of reaction.


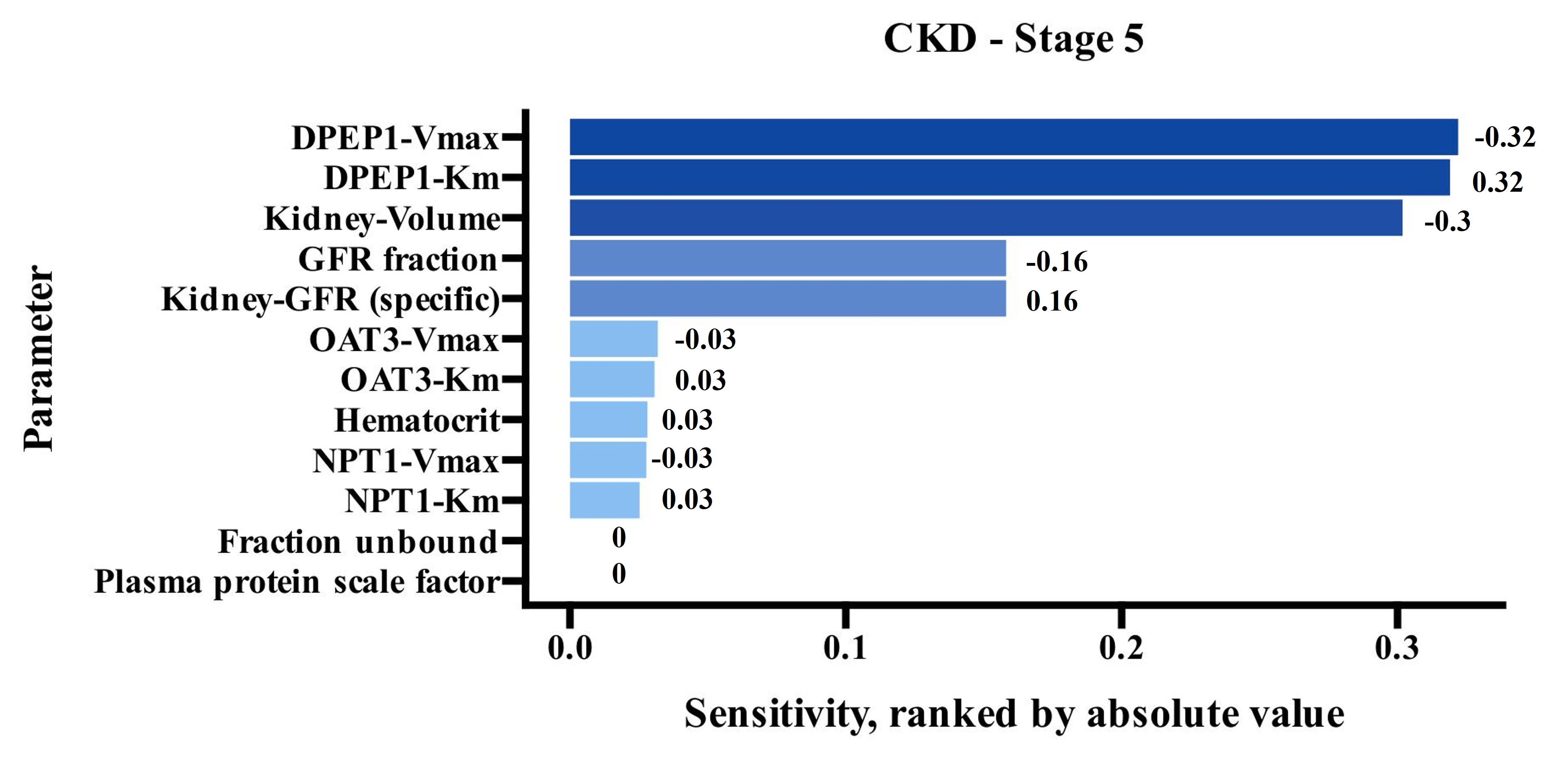


Supplementary Figure S6: Meropenem PBPK model sensitivity analysis in CKD stage 5. Sensitivity to single parameters, measured as change of the simulated AUC_last_ of a 500 mg intravenous infusion for 30min. Km, Michaelis–Menten constant; Vmax, maximum rate of reaction.


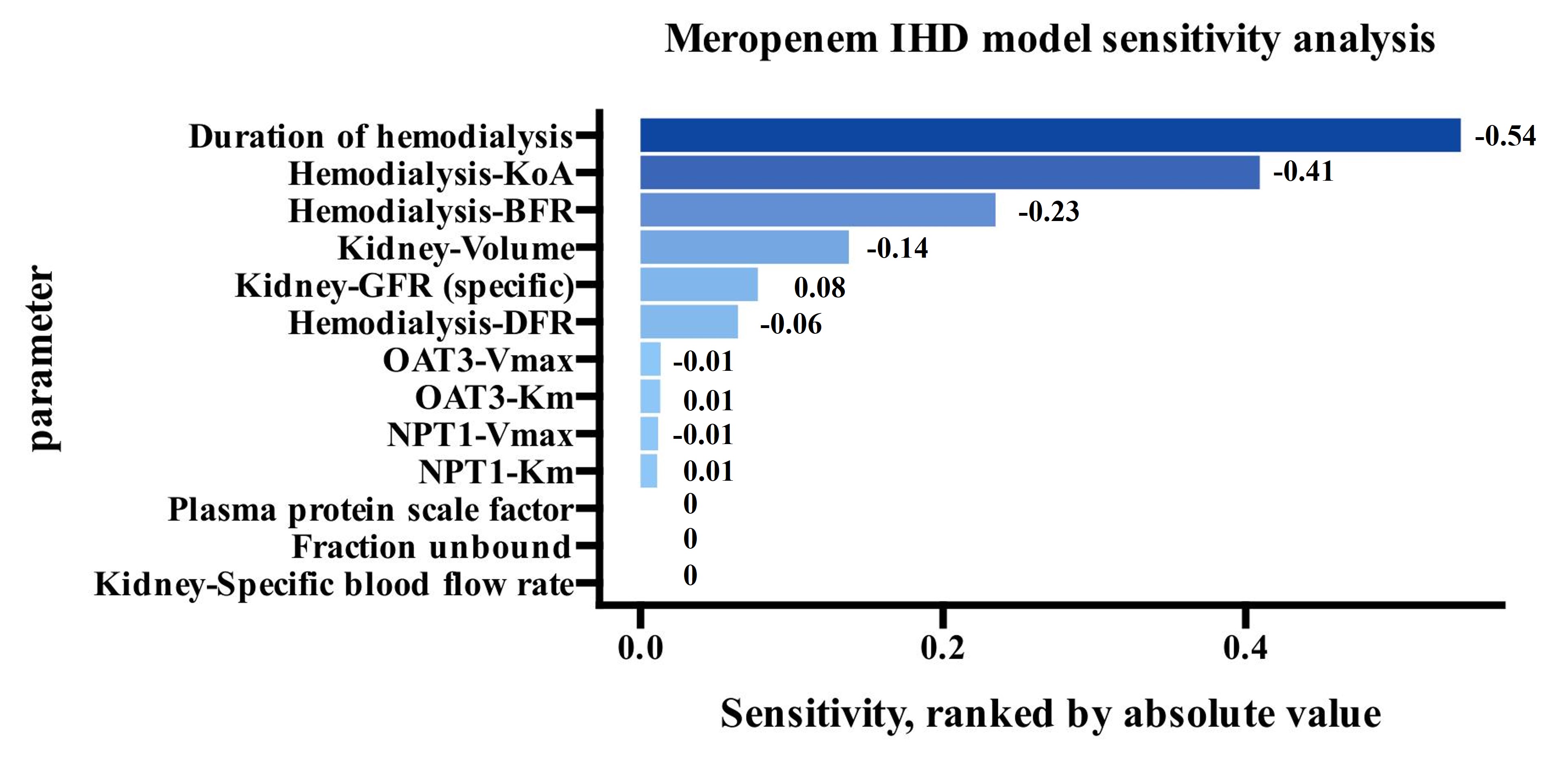


Supplementary Figure S7: Meropenem IHD-PBPK model sensitivity analysis. Sensitivity to single parameters, measured as change of the simulated AUC_last_ of a 500 mg intravenous infusion for 30min. Km, Michaelis–Menten constant; Vmax, maximum rate of reaction.

# Reference

Bax, R., Bastain, W., Featherstone, A., Wilkinson, D., and Hutchison, M. (1989). The pharmacokinetics of meropenem in volunteers. *Journal of antimicrobial chemotherapy* 24(suppl_A)**,** 311-320.

Chimata, M., Nagase, M., Suzuki, Y., Shimomura, M., and Kakuta, S. (1993). Pharmacokinetics of meropenem in patients with various degrees of renal function, including patients with end-stage renal disease. *Antimicrobial agents chemotherapy* 37(2)**,** 229-233.

Christensson, B., Nilsson-Ehle, I., Hutchison, M., Haworth, S., Oqvist, B., and Norrby, S. (1992). Pharmacokinetics of meropenem in subjects with various degrees of renal impairment. *Antimicrobial agents chemotherapy* 36(7)**,** 1532-1537.

Dandekar, P.K., Maglio, D., Sutherland, C.A., Nightingale, C.H., and Nicolau, D.P. (2003). Pharmacokinetics of meropenem 0.5 and 2 g every 8 hours as a 3‐hour infusion. *Pharmacotherapy: The Journal of Human Pharmacology Drug Therapy* 23(8)**,** 988-991.

Dreetz, M., Hamacher, J., Eller, J., Borner, K., Koeppe, P., Schaberg, T., et al. (1996). Serum bactericidal activities and comparative pharmacokinetics of meropenem and imipenem-cilastatin. *Antimicrobial agents chemotherapy* 40(1)**,** 105-109.

Harrison, M., Haworth, S., Moss, S., Wilkinson, D., and Featherstone, A. (1993). The disposition and metabolic fate of 14C-meropenem in man. *Xenobiotica* 23(11)**,** 1311-1323.

Leroy, A., Fillastre, J., Etienne, I., Borsa-Lebas, F., and Humbert, G. (1992). Pharmacokinetics of meropenem in subjects with renal insufficiency. *European journal of clinical pharmacology* 42(5)**,** 535-538.

Ljungberg, B., and Nilsson-Ehle, I. (1992). Pharmacokinetics of meropenem and its metabolite in young and elderly healthy men. *Antimicrobial agents chemotherapy* 36(7)**,** 1437-1440.

Nilsson-Ehle, I., Hutchison, M., Haworth, S., and Norrby, S. (1991). Pharmacokinetics of meropenem compared to imipenem-cilastatin in young, healthy males. *European Journal of Clinical Microbiology Infectious Diseases* 10(2)**,** 85-88.

Rubino, C.M., Bhavnani, S.M., Loutit, J.S., Lohse, B., Dudley, M.N., and Griffith, D.C. (2018a). Single-dose pharmacokinetics and safety of meropenem-vaborbactam in subjects with chronic renal impairment. *Antimicrobial Agents Chemotherapy* 62(3)**,** e02103-02117.

Rubino, C.M., Bhavnani, S.M., Loutit, J.S., Morgan, E.E., White, D., Dudley, M.N., et al. (2018b). Phase 1 study of the safety, tolerability, and pharmacokinetics of vaborbactam and meropenem alone and in combination following single and multiple doses in healthy adult subjects. *Antimicrobial Agents Chemotherapy* 62(4)**,** e02228-02217.

Wise, R., Logan, M., Cooper, M., Ashby, J., and Andrews, J. (1990). Meropenem pharmacokinetics and penetration into an inflammatory exudate. *Antimicrobial agents chemotherapy* 34(8)**,** 1515-1517.
